# Supplementary material for: Toxoplasmosis infection among pregnant women in Africa: A systematic review and meta-analysis
Source: PLoS One. 2021 Jul 20;16(7):e0254209. doi: 10.1371/journal.pone.0254209 (PMC8291666; doi:10.1371/journal.pone.0254209)
Supplement: S2 File — (DOCX) [file pone.0254209.s002.docx]

**S2 File: Sample search string of PubMed database.**

| Recent queries in PubMed | | |  |
| --- | --- | --- | --- |
| Search | Query | Items found | Time |
| #4 | Search ((Toxoplasmosis [Title/Abstract]) AND Pregnant Women [Title/Abstract]) AND Africa [Title/Abstract] | 19 | 12:20:51 |
| #3 | Search Africa [Title/Abstract] | 118288 | 12:16:07 |
| #2 | Search Pregnant Women [Title/Abstract] Sort by: [pubsolr12] | 89492 | 12:15:24 |
| #1 | Search Toxoplasmosis [Title/Abstract] Sort by: [pubsolr12] | 15498 | 12:14:10 |
