# Supplementary material for: Toxoplasmosis infection among pregnant women in Africa: A systematic review and meta-analysis
Source: PLoS One. 2021 Jul 20;16(7):e0254209. doi: 10.1371/journal.pone.0254209 (PMC8291666; doi:10.1371/journal.pone.0254209)
Supplement: S3 File — (DOCX) [file pone.0254209.s003.docx]

**Finding reported articles total 23 studies in this meta-analysis and systematic review. From different database likes: Embase, PubMed, AJOL, Scopus, Hinari and web of sciences**

Abamecha, F., & Awel, H. (2016). Seroprevalence and risk factors of Toxoplasma gondii infection in pregnant women following antenatal care at Mizan Aman General Hospital, Bench Maji Zone (BMZ), Ethiopia. *BMC Infect Dis, 16*(1), 460. doi:10.1186/s12879-016-1806-6

Agmas, B., Tesfaye, R., & Koye, D. N. (2015). Seroprevalence of Toxoplasma gondii infection and associated risk factors among pregnant women in Debre Tabor, Northwest Ethiopia. *BMC Res Notes, 8*(1), 107. doi:10.1186/s13104-015-1083-2

Awoke, K., Nibret, E., & Munshea, A. (2015). Sero-prevalence and associated risk factors of Toxoplasma gondii infection among pregnant women attending antenatal care at Felege Hiwot Referral Hospital, northwest Ethiopia. *Asian Pacific journal of tropical medicine, 8*(7), 549-554.

Bamba, S., Cissé, M., Sangaré, I., Zida, A., Ouattara, S., & Guiguemdé, R. T. (2017). Seroprevalence and risk factors of Toxoplasma gondii infection in pregnant women from Bobo Dioulasso, Burkina Faso. *BMC Infect Dis, 17*(1), 482-486. doi:10.1186/s12879-017-2583-6

De Paschale, M., Ceriani, C., Cerulli, T., Cagnin, D., Cavallari, S., Cianflone, A., . . . Zaongo, D. (2014). Antenatal screening for Toxoplasma gondii, Cytomegalovirus, rubella and Treponema pallidum infections in northern Benin. *Tropical Medicine & International Health, 19*(6), 743-746.

Doudou Yobi , Renaud Piarroux, Coralie L’Ollivier, Jacqueline Franck, Hypolite Situakibanza, Hypolite Muhindo, . . . Lutumba. (2014). Toxoplasmosis among pregnant women:High seroprevalence and risk factors in Kinshasa,Democratic Republic of Congo. *亚太热带生物医学杂志：英文版, 4*(1), 69-74. doi:10.1016/S2221-1691(14)60211-2

Endris, M., Belyhun, Y., Moges, F., Adefiris, M., Tekeste, Z., Mulu, A., & Kassu, A. (2014). Seroprevalence and Associated Risk Factors of Toxoplasma gondii in Pregnant Women Attending in Northwest Ethiopia. *Iranian journal of parasitology, 9*(3), 407-414.

Fenta, D. A. (2019). Seroprevalence of Toxoplasma gondii among pregnant women attending antenatal clinics at Hawassa University comprehensive specialized and Yirgalem General Hospitals, in Southern Ethiopia. *BMC Infect Dis, 19*(1), 1056-1059. doi:10.1186/s12879-019-4694-8

Frimpong, C., Makasa, M., Sitali, L., & Michelo, C. (2017). Seroprevalence and determinants of toxoplasmosis in pregnant women attending antenatal clinic at the university teaching hospital, Lusaka, Zambia. *BMC Infect Dis, 17*(1), 10. doi:10.1186/s12879-016-2133-7

Gelaye, W., Kebede, T., & Hailu, A. (2015). High prevalence of anti-toxoplasma antibodies and absence of Toxoplasma gondii infection risk factors among pregnant women attending routine antenatal care in two Hospitals of Addis Ababa, Ethiopia. *International Journal of Infectious Diseases, 34*(C), 41-45. doi:10.1016/j.ijid.2015.03.005

Koffi, M., Konaté, I., Sokouri, D., Konan, T., Ahouty, B., & Bosso, J. (2015). Seroepidemiology of Toxoplasmosis in Pregnant Women Attending Antenatal Clinics at the Center for Maternal and Child Health Care in Daloa in Ivory Coast. *International Journal of Tropical Disease & Health, 6*(4), 125-132.

Linguissi, L. S. G., Nagalo, B. M., Bisseye, C., Kagoné, T. S., Sanou, M., Tao, I., . . . Koné, B. (2012). Seroprevalence of toxoplasmosis and rubella in pregnant women attending antenatal private clinic at Ouagadougou, Burkina Faso. *Asian Pacific journal of tropical medicine, 5*(10), 810-813.

Murebwayire, E., Njanaake, K., Ngabonziza, J. C. S., Jaoko, W., & Njunwa, K. J. (2017). Seroprevalence and risk factors of Toxoplasma gondii infection among pregnant women attending antenatal care in Kigali, Rwanda. *Tanzania journal of health research, 19*(1).

Mwambe, B., Mshana, S. E., Kidenya, B. R., Massinde, A. N., Mazigo, H. D., Michael, D., . . . Groß, U. (2013). Sero-prevalence and factors associated with Toxoplasma gondii infection among pregnant women attending antenatal care in Mwanza, Tanzania. *Parasites & vectors, 6*(1), 222-222. doi:10.1186/1756-3305-6-222

Nasir, I. A., Aderinsayo, A. H., Mele, H. U., & Aliyu, M. M. (2015). Prevalence and associated risk factors of Toxoplasma gondii antibodies among pregnant women attending Maiduguri teaching hospital, Nigeria. *Journal of Medical Sciences, 15*(3), 147.

Negero, J., Yohannes, M., Woldemichael, K., & Tegegne, D. (2017). Seroprevalence and potential risk factors of T. gondii infection in pregnant women attending antenatal care at Bonga Hospital, Southwestern Ethiopia. *International Journal of Infectious Diseases, 57*(C), 44-49. doi:10.1016/j.ijid.2017.01.013

Njunda, A. L., Assob, J. C. N., Nsagha, D. S., Kamga, H. L. F., Nde, P. F., & Yugah, V. C. (2011). Seroprevalence of Toxoplasma gondii infection among pregnant women in Cameroon. *Journal of Public Health in Africa, 2*(2), 24. doi:10.4081/jphia.2011.e24

Olusi, T., Grob, U., & Ajayi, J. (1996). High incidence of toxoplasmosis during pregnancy in Nigeria. *Scandinavian journal of infectious diseases, 28*(6), 645-646.

Paul, E., Kiwelu, I., Mmbaga, B., Nazareth, R., Sabuni, E., Maro, A., . . . Chilongola, J. (2018). Toxoplasma gondii seroprevalence among pregnant women attending antenatal clinic in Northern Tanzania. *Tropical medicine and health, 46*(1), 39-38. doi:10.1186/s41182-018-0122-9

Rodier, M. H., Berthonneau, J., Bourgoin, A., Giraudeau, G., Agius, G., Burucoa, C., . . . Jacquemin, J. L. (1995). Seroprevalences of Toxoplasma, malaria, rubella, cytomegalovirus, HIV and treponemal infections among pregnant women in Cotonou, Republic of Benin. *Acta Trop, 59*(4), 271-277. doi:10.1016/0001-706x(95)00087-u

Simpore, J., Savadogo, A., Ilboudo, D., Nadambega, M. C., Esposito, M., Yara, J., . . . Musumeci, S. (2006). Toxoplasma gondii, HCV, and HBV seroprevalence and co‐infection among HIV‐positive and‐negative pregnant women in Burkina Faso. *Journal of medical virology, 78*(6), 730-733.

Teweldemedhin, M., Gebremichael, A., Geberkirstos, G., Hadush, H., Gebrewahid, T., Asgedom, S. W., . . . Gebreyesus, H. (2019). Seroprevalence and risk factors of Toxoplasma gondii among pregnant women in Adwa district, northern Ethiopia. *BMC Infect Dis, 19*(1), 327-329. doi:10.1186/s12879-019-3936-0

Zemene, E., Yewhalaw, D., Abera, S., Belay, T., Samuel, A., & Zeynudin, A. (2012). Seroprevalence of Toxoplasma gondii and associated risk factors among pregnant women in Jimma town, Southwestern Ethiopia. *BMC Infect Dis, 12*(1), 337-337. doi:10.1186/1471-2334-12-337
