# Supplementary material for: Toxoplasmosis infection among pregnant women in Africa: A systematic review and meta-analysis
Source: PLoS One. 2021 Jul 20;16(7):e0254209. doi: 10.1371/journal.pone.0254209 (PMC8291666; doi:10.1371/journal.pone.0254209)
Supplement: Attachment — Submitted filename: Response to reviewer #1.docx [file pone.0254209.s005.docx]

**Author response to reviewer**

1. Reviewer #1:

1- In line 41 You have indicated that you have presented the Odds ratio with a 95% confidence interval of meta-analysis the random effect model for your pooled prevalence however the Odds ratio have been mentioning in your abstract only and nothing about it in any part of your text. You need to elaborate on the clearly in your manuscript text.

**Answer**

**We have mentioned odd ration and 95%CI in table 2. Page on 13. This question was corrected based on comments.**

2- It good that the authors have included 23 studies in the analysis however the authors could discuss and reveal the biases, strengths, and weaknesses of those included studies.

Answer

We tried to address all papers strength and weakness before included in analysis.

Only weakness of papers by design types mentioned. The rest things should report by authors about articles strength. But not important for my analysis to write the papers strength and weakness.

3- In several places throughout the characteristics of included studies, prevalence of toxoplasmosis of pregnant women in Africa and meta-regression analysis for sources of heterogeneity sections, " 4 serological analysis tests" has been mentioned as a method to test the presence of anti- Toxoplasmosis gondii antibodies. However, nothing has been mentioned about specificity or sensitivity of these tests or what is the source of differences between them. I suggest the authors could elaborate on that more. This increases the clarity of the sources of heterogeneity.

**Answer.**

Really, I appreciated you comment on specificity and sensitivity issues. But all primary studies were not mentioned its sensitivity and specificity of each articles. Simple the authors have reported as the types of tests. That why we have used to check source of the heterogeneity for papers.

4- Meta-regression analysis for sources of heterogeneity and discussion give little information on the heterogeneity of pooled prevalence. It will be more useful if the authors can provide more details on source for heterogeneity and reveal the biases, strengths, and weaknesses of existing studies.

Answer

This question was corrected by above questions.

5- The authors have been able to make a balance between finding of the included studies that are similar and appropriate to combine without becoming too focused, also authors have been able to avoid a study population that is too narrow. However, the author needs to elaborate more on validity and reliability of their study inference taking in consideration that the other studies written not by the English language were not considered in this analysis thin the studies from the northern and southern region of Africa not included. Please address the generalizable in more detail.

**Answer**

**Once again, we appreciated you detail insight of this paper. Above point mentioned as weakness of meta-analysis. we lack of article from norther and southern region of Africa.**

**Thank you for you detail comments our reviewers**
